# *Toxoplasma gondii* Infection Is Associated with Mitochondrial Dysfunction *in-Vitro*

**DOI:** 10.3389/fcimb.2017.00512

**Published:** 2017-12-12

**Authors:** Genevieve Syn, Denise Anderson, Jenefer M. Blackwell, Sarra E. Jamieson

**Affiliations:** Genetics and Health, Telethon Kids Institute, University of Western Australia, Subiaco, WA, Australia

**Keywords:** *Toxoplasma gondii*, mitochondria dysfunction, oxidative phosphorylation, gene expression profiling, membrane potential, mitochondrial superoxide, congenital toxoplasmosis, latent toxoplasmosis

## Abstract

Upon invasion of host cells, the ubiquitous pathogen *Toxoplasma gondii* manipulates several host processes, including re-organization of host organelles, to create a replicative niche. Host mitochondrial association to *T. gondii* parasitophorous vacuoles is rapid and has roles in modulating host immune responses. Here gene expression profiling of *T. gondii* infected cells reveals enrichment of genes involved in oxidative phosphorylation (OXPHOS) and mitochondrial dysfunction 6 h post-infection. We identified 11 hub genes (*HIF-*1α, *CASP8, FN1, POU5F1, CD44, ISG15, HNRNPA1, MDM2, RPL35, VHL*, and *NUPR1*) and 10 predicted upstream regulators, including 4 endogenous regulators RICTOR, KDM5A, RB1, and D-glucose. We characterized a number of mitochondrial parameters in *T. gondii* infected human foreskin fibroblast cells over a 36 h time-course. In addition to the usual rapid recruitment and apparent enlargement of mitochondria around the parasitophorous vacuole we observed fragmented host mitochondria in infected cells, not linked to cellular apoptosis, from 24 h post-infection. An increase in mitochondrial superoxide levels in *T. gondii* infected cells was observed that required active parasite invasion and peaked at 30 h post-infection. Measurement of OXPHOS proteins showed decreased expression of Complex IV in infected cells at 24 h post-infection, followed by decreased expression of Complexes I and II at 36 h post-infection. No change occurred in Complex V. No difference in host mitochondrial membrane potential between infected and mock-infected cells was observed at any time. Our results show perturbation of host mitochondrial function following *T. gondii* infection that likely impacts on pathogenesis of disease.

## Introduction

Mitochondria are powerhouses of the cell, generating most of the cellular energy in the form of adenosine triphosphate (ATP) through oxidative phosphorylation (OXPHOS). This process occurs at the electron transport chain found on the inner mitochondrial membrane comprising of the OXPHOS complexes. Complex I to IV are four multi-subunit enzymes working together to create an electrochemical proton gradient across the mitochondrial inner membrane. ATP synthase (Complex V) then uses the proton gradient to generate ATP. In addition to their central role in metabolism, mitochondria also regulate cellular processes such as cell cycle (Antico Arciuch et al., 2012), innate immunity (West et al., 2011), and apoptosis (Wang and Youle, 2009). During an infection with the ubiquitous pathogen *Toxoplasma gondii*, these same processes are modulated in the host cell suggesting a central role for host mitochondria in establishing infection in host cells. *T. gondii* infection is common in man, although most infections are asymptomatic. However, congenital disease arising from vertical transmission following primary infection during pregnancy, or acquired infection in immunocompromised individuals, can result in a range of debilitating and potentially fatal ocular and/or brain lesions. The mechanisms leading to these clinical signs in both acquired and congenital toxoplasmosis remain poorly understood.

Upon invasion of the host cell, *T. gondii* must manipulate several of its host's processes to survive and replicate. It dysregulates the host cell cycle (Molestina et al., 2008), inhibits mitochondrial-dependent host apoptosis (Goebel et al., 2001; Lüder and Gross, 2005), subverts the host innate immune system (Lambert and Barragan, 2010) and recruits host mitochondria to the parasitophorous vacuole (de Melo et al., 1992; Lindsay et al., 1993; Sinai et al., 1997; Pernas et al., 2014) following infection. Association of the mitochondria to *T. gondii*'s parasitophorous vacuole membrane (PVM) occurs as quickly as 10 min after invasion (Sinai and Joiner, 2001). Contact between the outer mitochondrial membrane and PVM was observed to be continuous over the length of the mitochondrial profile and the extent of PVM-mitochondria association did not change over time even as the PVM grew larger to accommodate increased numbers of parasites (Sinai et al., 1997). The PVM-associated mitochondria are morphologically distinct from cytosolic mitochondria, labeling more intensely with membrane potential sensitive dyes (Sinai et al., 1997) and with cross-sections that are ~3-fold larger than cytosolic mitochondria when measured at 18 h post-infection (Pernas et al., 2014). PVM-mitochondria association is also parasite strain-specific; the mouse-virulent type I and III strains show host mitochondrial association while the avirulent type II strain does not (Pernas et al., 2014) suggesting that recruitment of host mitochondria may have a role to play in determining pathogenicity and the severity of clinical signs associated with toxoplasmosis.

The importance of mitochondria during *T. gondii* infection has been further highlighted in a study carried out by Nelson et al. (2008) characterizing host cell proteomic changes following *T. gondii* infection which showed one-third of modulated proteins were mitochondrial. Initially hypothesized to be for the acquisition of nutrients, such as glucose and amino acids unable to be synthesized by *T. gondii* (Sinai et al., 1997), PVM-mitochondrial association has also been linked to the modulation of the innate immune response (Pernas et al., 2014).

Given *T. gondii*'s intricate relationship with the host mitochondria, we wanted to understand in more detail what the effects of this PVM-mitochondrial association were on host mitochondrial function. Here we present results of a microarray study that shows OXPHOS and mitochondrial pathways as the top canonical pathways perturbed during infection of human foreskin fibroblast (HFF) cells with *T. gondii* RH strain (type I). We further characterize the effect of *T. gondii* on mitochondrial function by demonstrating modulation of the host mitochondrial morphology, superoxide production, and OXPHOS protein expression over time, pointing to a perturbation of mitochondrial function. We also highlight similarities between the clinical signs of patients suffering from mitochondrial dysfunction and toxoplasmosis.

## Materials and methods

### Parasite and cell culture

Primary Human Foreskin Fibroblast (HFF) monolayers were cultured in Dulbecco's Modified Eagle's Medium (DMEM) (Gibco®, Life Technologies) supplemented with 2 mM GlutaMAX^TM^ (Gibco®, Life Technologies), 100 U/ml Penicillin/100 μg/ml Streptomycin (Gibco®, Life Technologies) and 10% heat-inactivated fetal calf serum (Gibco®). Wild-type RH type I strain *T. gondii* tachyzoites (gift from Dr Tanya Armstrong, Murdoch University) and green fluorescent protein (GFP)- and mCherry-expressing RH type I strain *T. gondii* tachyzoites (gift from Dr. Chris Tonkin, Walter and Eliza Hall Institute of Medical Research) were maintained *in-vitro* in HFF monolayers grown in infection medium (DMEM supplemented as previously described but with 1% heat-inactivated fetal calf serum). All cultures were grown in a 37°C humidified CO_2_ (5%) incubator. Continuous passage of the parasites was carried out by harvesting infected HFF monolayer with a cell scraper (Sarstedt) and passing it twice through a 27 gauge needle (Becton) to forcibly rupture and release the parasites from any intact infected HFF cells. Host cell debris was removed by passing the parasite suspension through 5 micron pore Millex® syringe filter units (Merck Millipore). The filtered parasites were then used to infect other cell monolayers. In experimental studies, mock-infected cells were used as controls, i.e., cells treated with media containing uninfected cells harvested and treated through the same processes as when harvesting and purifying parasites. Microscopic counts of parasites and flow cytometry was used to demonstrate infection rates and survival of GFP-expressing (44.7% ± 10.3) and mCherry-expressing (61.3% ± 4.3) and untransfected wild-type RH strain tachyzoites (30.2% ± 7.9).

### RNA extractions

HFF cells (1 × 10^6^) were seeded into each well of a six-well tissue culture treated plate (Becton) and infected with wild type *T. gondii* at a multiplicity of infection (MOI) of 10:1. Two biological replicates were carried out for each experimental condition. Two hours after addition of parasites, cells were washed twice with infection medium, to remove any extracellular parasites and the medium replaced with fresh infection medium to minimize continued *T. gondii* invasion at later time points. Mock-infected HFF cells were prepared in the same way as infected cells but treated with media prepared as described above. Cells were harvested at 2, 6, and 24 h post-infection using Tri-reagent® (Sigma-Aldrich) and total RNA was extracted using Direct-Zol^TM^ RNA Miniprep kit (Zymo Research) following manufacturer's instructions.

### Gene expression profiling and analysis

Generation of microarray data on the Illumina HumanHT-12 v4 Expression Beadchips was carried out at the Australian Genome Research Facility (Melbourne node, Australia). All data analysis was carried out in R Version 3.1.3 (Smooth Sidewalk - https://www.r-project.org/) and RStudio (version 0.99.484). The Bioconductor package *lumi* (Du et al., 2008) was used to read in raw expression values and perform quality control, background correction and normalization of the data. Pre-processing of the microarray data and removal of probes previously shown to have unreliable annotation (Barbosa-Morais et al., 2010) resulted in 28,584 probes which passed QC requirements. The data discussed in this manuscript have been deposited in NCBI's Gene Expression Omnibus (Edgar et al., 2002) and are accessible through GEO Series accession number GSE98677 (https://www.ncbi.nlm.nih.gov/geo/query/acc.cgi?acc=GSE98677). Differential expression analysis using linear modeling and empirical Bayes methods was carried out in the Bioconductor package *limma* (Ritchie et al., 2015) with comparison of infected cells vs. mock-infected controls at each time point. Following Benjamini-Hochberg correction (Benjamini and Hochberg, 1995) for multiple testing, genes with an adjusted *p*-value < 0.05 and a fold change of ≥ 1.5 formed the top sets of genes taken forward into *in-silico* pathway and network analyses.

### Bioinformatics analyses of differentially expressed genes

Ingenuity Pathway Analysis (IPA) (Ingenuity Systems, www.ingenuity.com) was used to identify canonical pathways associated with the differentially expressed genes. IPA uses the Ingenuity Knowledge Base, an extensive database comprising of biological pathways and functional annotations derived from the interactions between genes, proteins, complexes, drugs, tissues, and disease, to carry out all its analyses. Benjamini-Hochberg correction was applied where appropriate and an adjusted *p*-value < 0.05 was used to filter all results. Upstream Regulator Analysis within IPA was employed to predict if there were any upstream regulators, including transcription factors, chemicals and drugs/compounds, which may be responsible for the observed gene expression patterns. A *p*-value < 0.05 was used to determine significance. If an upstream regulator is identified, an activation z-score is calculated based on the fold change values of its target genes within the dataset. A z-score ≥ 2 suggests that an upstream regulator is activated, whereas a z-score ≤ −2 suggests it is inhibited, in our experimental group vs. the control. In all of our analyses the mock-infected cells formed the baseline comparator. Networks were constructed in IPA by selecting all genes within each time point and using the “Connect” option under the “Build” functionality. Genes with no previously documented interactions were removed from the diagram and the functions of each network were inferred from the remaining connected genes at each time point.

### Assessment of mitochondrial function

All mitochondrial function assays were performed using HFF cells infected with either wild-type, mCherry- or GFP-expressing *T. gondii* tachyzoites, as indicated, at a MOI of 10:1. Two hours after addition of parasites, the cells were washed twice with warmed sterile Hank's Balanced Salt Solution (HBSS) (Sigma-Aldrich) and the medium replaced with fresh infection media to minimize continued *T. gondii* invasion. Cells were harvested over a time-course of 6, 12, 18, 24, 30, and 36 h post-infection. Mock-infected HFF cells (as previously described) were used as controls.

#### Analysis of mitochondrial morphology

Cells were seeded onto 13 mm round glass coverslips #1 thickness (ProSciTech) and infection was carried out using GFP-expressing parasites. Mitochondria in live intact cells were stained with 500 nM MitoTracker™ Orange CMTRos (Life Technologies) in infection medium in a 37°C humidified CO_2_ (5%) incubator for 45 min, followed by nuclear staining with NucBlue ® Live ReadyProbes ® Reagent (Life Technologies) for 10 min. Cells were fixed with 4% paraformaldehyde (Sigma-Aldrich) for 20 min, washed thrice with Phosphate Buffered Saline (PBS) (Medicago, Astral Scientific) and mounted onto glass slides with Shanndon^TM^ Immu-Mount^TM^ (Thermo Fisher Scientific) before visualization under Nikon C2+ Confocal Microscopy at 60X magnification under oil immersion. Black and white images of mitochondria, GFP-expressing parasites and nucleus captured in different channels were imported into ImageJ (Schneider et al., 2012) and overlaid with colors red, green, and blue respectively. Colors were optimized to similar intensities and the images merged to form a composite figure. Three biological experiments were performed. To determine the extent of mitochondrial fragmentation, parasitophorous vacuoles were quantified from these composite images for intact or disrupted mitochondria at each time-point. Host mitochondria surrounding the parasitophorous vacuole as one continuous structure were classed as “intact” and host mitochondria surrounding the parasitophorous vacuole in segments were classed as “disrupted”.

#### Annexin V

Infection was carried out in 24-well plates with non-fluorescent wild-type parasites. Cells were dislodged using TrypLE^TM^ Express and re-suspended in 100 μl of 1X Annexin V Buffer (Life Technologies) containing 5 μl of Annexin V Alexa Fluor 647 conjugate (Life Technologies). Cells were incubated in a 37°C humidified CO_2_ (5%) incubator for 15 min. Annexin V staining was determined using flow cytometry and the data were processed in FlowJo^TM^ (v10) (Treestar, USA). The number of Annexin V positive cells was exported into a spreadsheet, plotted and statistical analysis performed as per section Statistical Analyses. This experiment was done once with three technical replicates.

#### Measurement of mitochondrial superoxide

Infection was carried out in 24-well plates (50,000 cells/well) using GFP-expressing parasites. Two biological experiments were carried out with three technical replicates per biological experiment. Cells were stained with 5 μM of MitoSOX^TM^ Red (Life Technologies) in infection medium in a 37°C humidified CO_2_ (5%) incubator for 30 min. As a positive control, cells were treated with 10 μM antimycin-A (Sigma-Aldrich) during staining to generate mitochondrial ROS. Cells were washed once with warmed HBSS and dislodged using TrypLE^TM^ Express. After centrifugation at 1,000 × g for 5 min at 10°C, cells were re-suspended in HBSS FACS Buffer [HBSS + 5 mM EDTA (Ambion®, ThermoFisher Scientific) + 1% Bovine Serum Albumin (BSA) (Sigma-Aldrich)]. MitoSOX^TM^ fluorescence was detected using flow cytometry (BD LSRFortessa^TM^) and the data processed using FlowJo® (v10). Forward scatter (FSC) and side scatter (SSC) parameters were used to exclude cellular debris, dead cells, and doublets to retain viable single cell events. As GFP-expressing parasites were used in this experiment, infected cells (GFP-positive) could be differentiated from uninfected cells (GFP-negative) in the same population. When GFP parasites replicate within a cell, the GFP fluorescent intensity of infected cells increases over time. Since we were interested in the dynamics of superoxide production in infected cells over time, we segregated cells situated at the lower end of the GFP fluorescent intensity spectrum (newly-infected cells) from those at the higher end (cells with an established infection) in later time points. The median fluorescent intensities of MitoSOX^TM^ in each treatment group (established infection, newly infected, uninfected, and mock-infected) were exported into a spreadsheet, values plotted, and statistical analyses performed as described in section Statistical Analyses.

#### OXHPOS western blot analyses

Infection was carried out using mCherry-expressing parasites. At each time point post-infection, cells were dislodged using TrypLE^TM^ Express (Life Technologies) and infected (mCherry positive) and uninfected (mCherry negative) cells were separated using a cell sorter (BD FACS Aria III sorter). Infected cells were pelleted at 6,000 × g for 3 min and total protein was extracted using Radioimmunoprecipitation Assay (RIPA) buffer. Proteins were quantified with Direct-Detect® Infrared Spectrometer (Merck Millipore). NuPAGE® LDS Sample Buffer (4X) (Life Technologies). NuPAGE® Sample Reducing Agent (10X) (Life Technologies) was added to 20 μg of protein per sample as per manufacturer's instructions and incubated at 37°C for 30 min before being quenched on ice for at least 2 min. Proteins were separated by gel electrophoresis on NuPAGE Bis-Tris 4-12% protein gels (Life Technologies) and blotted onto Polyvinylidene fluoride (PVDF) membranes. Membranes were blocked with 5% skim milk for an hour in Tris Buffered Saline with 0.1% Tween-20 (Sigma-Aldrich) (TBST) prior to incubation at 4°C overnight with mouse anti-OXPHOS antibody cocktail (1:1,000; ab110413; Abcam) and mouse monoclonal anti-β-actin antibody (1:5,000; A1978–200 μl; Sigma–Aldrich) as a loading control. Secondary staining with horseradish peroxidase-conjugated sheep anti-mouse antibodies (1:10,000; NA931–100UL; GE Healthcare) was carried out for at least 2 h before chemiluminescence detection. Following addition of Amersham ECL Western Blotting Detection Reagent (GE Healthcare), imaging of the PVDF membranes were done with the Biorad ChemiDoc^TM^ MP imaging system.

The difference in band intensities between treatment groups was quantified using ImageJ (Schneider et al., 2012). After importing the membrane images into ImageJ, the bands to be analyzed were selected using the “Rectangular Select” tool and presented as histograms representing the relative density of the bands. To remove background noise, a line was drawn across the bottom of the histogram with the “Straight Line” tool to enclose the peak. The area under the curve was translated into a numerical value by clicking inside the peak using the “Wand (tracing)” tool; the brighter the band the higher the value. These values were then imported into a spreadsheet and all band intensities of the OXPHOS complexes were normalized to the loading control β-actin. The normalized values of infected cells at each time point were then compared to their respective mock-infected cells, their mean percentage difference plotted, and statistical analyses performed as described in section Statistical Analyses. Three independent biological experiments were performed.

#### Measurement of mitochondrial membrane potential

As the fluorescent emission of JC-1 overlapped with the emission spectra of GFP- and mCherry-expressing parasites, infection was carried out in 24-well plates (50,000 cells/well) with non-fluorescent wild-type parasites. Three independent biological experiments with three technical replicates each were performed. Cells were incubated with 2 μM of JC-1 (Life Technologies) in infection medium in a 37°C humidified CO_2_ (5%) incubator for 30 min. As a positive control, 50 μM of carbonyl cyanide *m*-chlorophenyl hydrazone (CCCP) (Life Technologies) was added to uninfected cells during staining to depolarise the mitochondrial membrane. Cells were washed once with warmed HBSS, dislodged using TrypLE^TM^ Express and re-suspended in 200 μl of HBSS. Changes in mitochondrial membrane potential were determined using flow cytometry and the data were processed in FlowJo^TM^ (v10). Cellular debris, dead cells, and doublets were excluded from the analysis. As non-fluorescent parasites were used in the infection experiments, we were unable to differentiate between infected and uninfected cells thus cells from the whole population were analyzed. Membrane potential was visualized as a ratio of red fluorescence (conjugated form of JC-1 due to negative membrane potential) to green fluorescence (unconjugated form of JC-1 due to positive membrane potential). Cells treated with CCCP were used to set the gates defining cells with depolarized mitochondrial membranes.

### Statistical analyses

All graphs and statistics were generated using GraphPad Prism version 7.00 for Windows (GraphPad Software, La Jolla California USA, www.graphpad.com). A two-way ANOVA test was performed to compare the population means of treatment groups and Bonferroni correction for multiple testing was applied. Comparisons with an adjusted *p*-value ≤ 0.05 were considered to be significant.

## Results

### *T. gondii* infection results in numerous differentially expressed genes at 6 H post-infection

**Figure 1:**
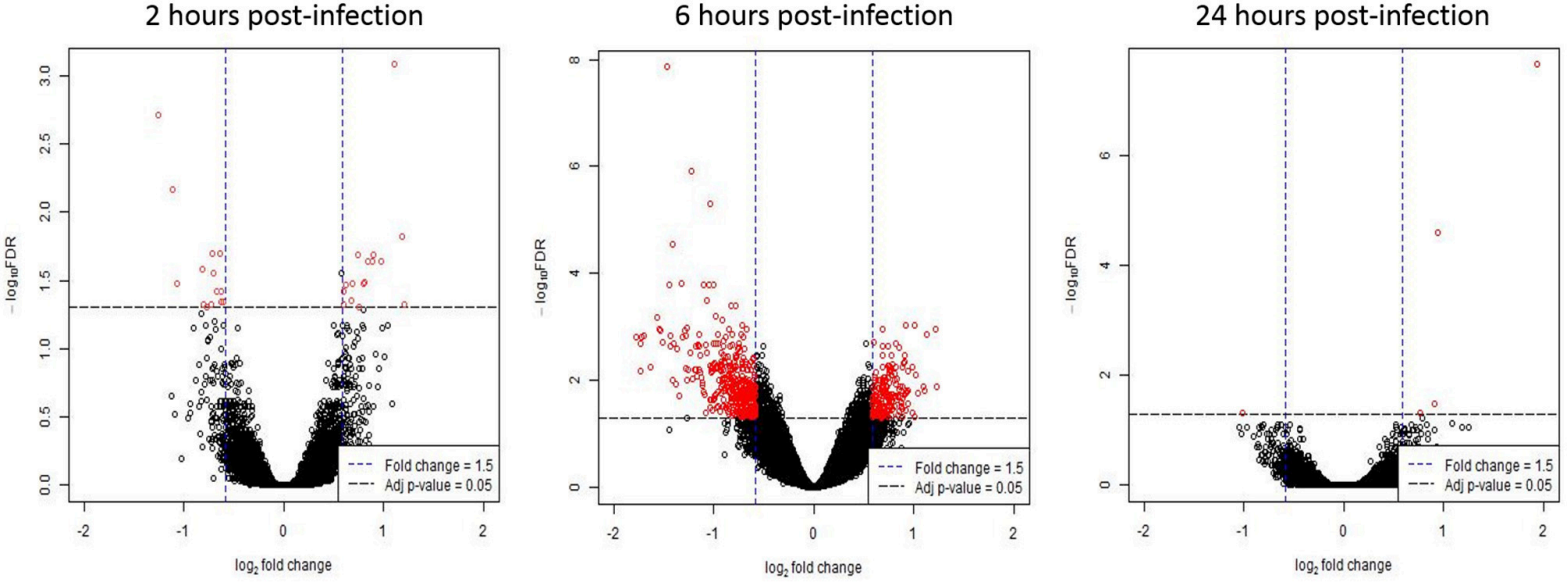
Differentially expressed genes following *T. gondii* infection. Results of tests for differential expression at each time point are presented in a volcano plot which plots statistical significance against fold change for each gene. Genes colored in red have Benjamini and Hochberg adjusted *p*-values of < 0.05 and absolute fold changes of ≥ 1.5 and are deemed of interest for *in-silico* pathway analyses.

Gene expression values of *T. gondii* infected HFF cells were compared against mock-infected HFF cells (baseline) at each time point. At 2 h post-infection, 30 genes were differentially expressed in infected cells; 16 genes had increased expression; and 14 genes had decreased expression (Supplementary Table 1). At 6 h post-infection, there were 498 differentially expressed genes; 172 genes had increased expression; and 326 genes had decreased expression following *T. gondii* infection (Supplementary Table 1). At 24 h post-infection, four genes were differentially expressed; three genes had increased expression and one gene had decreased expression in *T. gondii* infected cells (Supplementary Table 1). Figure 1 shows a volcano plot highlighting the 498 genes with an adjusted *p*-value of < 0.05 and an absolute fold change of ≥ 1.5 at 6 h post-infection. These genes were deemed of interest for *in-silico* pathway analyses.

### Network and pathway analyses of genes differentially expressed at 6 H post *T. gondii* infection reveals dysregulation of mitochondrial pathways

In order to understand more about the effect of *T. gondii* on the biology of host cells the data for differentially expressed genes at 6 h post-infection was analyzed using IPA. There were insufficient numbers of differentially expressed genes at 2 and 24 h post-infection for meaningful pathway analyses. Of the 498 differentially expressed genes at 6 h post-infection (6 h dataset), 495 genes (99%) mapped to their respective molecules within the Ingenuity Knowledge Base.

**Figure 2:**
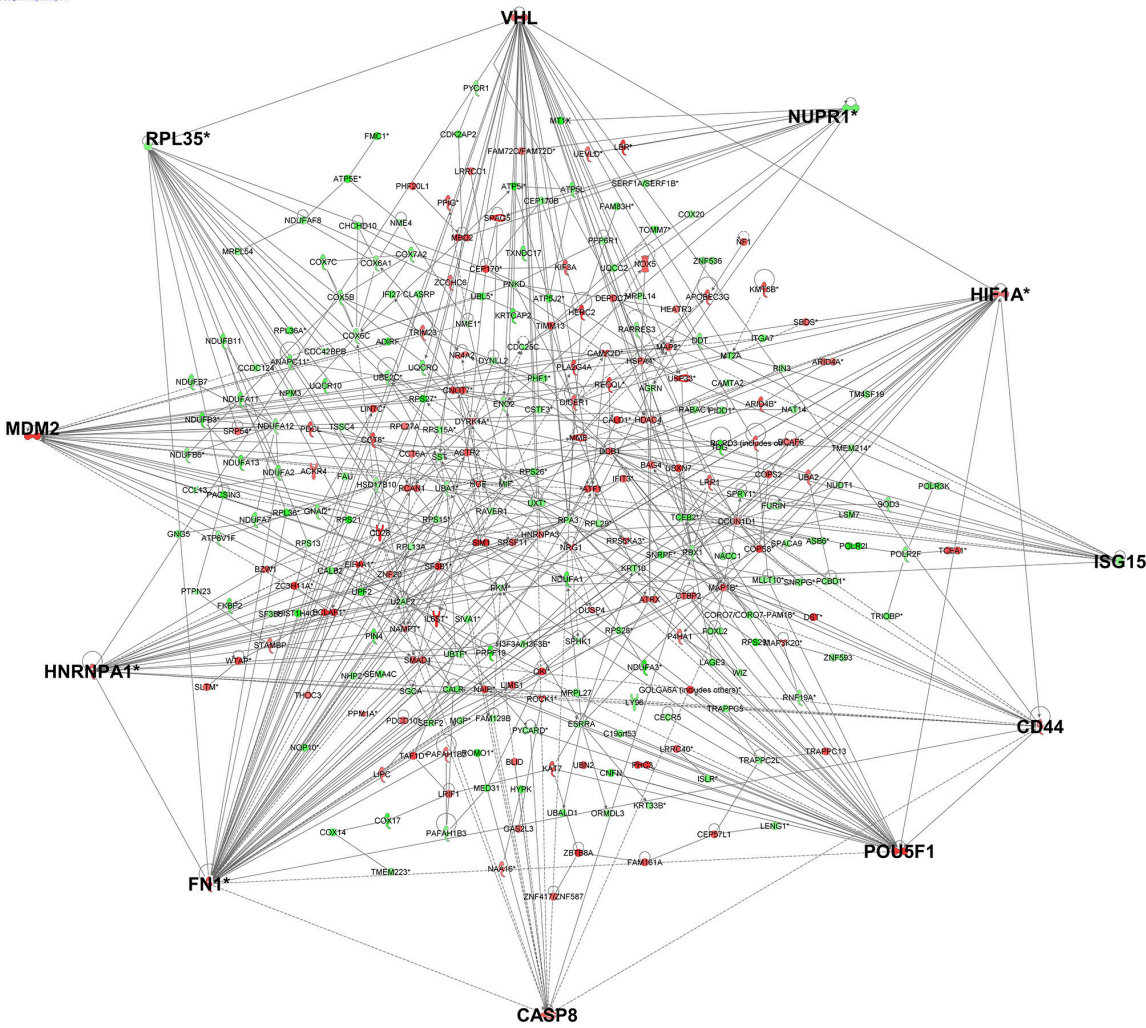
Gene networks generated in IPA for the genes differentially expressed in cells infected for 6 h identified 11 hub genes. Genes in red have increased expression and genes in green have decreased expression in *T. gondii* infected cells. The more intense the color, the higher the fold change values. Genes highlighted in bold are hub genes.

**Figure 3:**
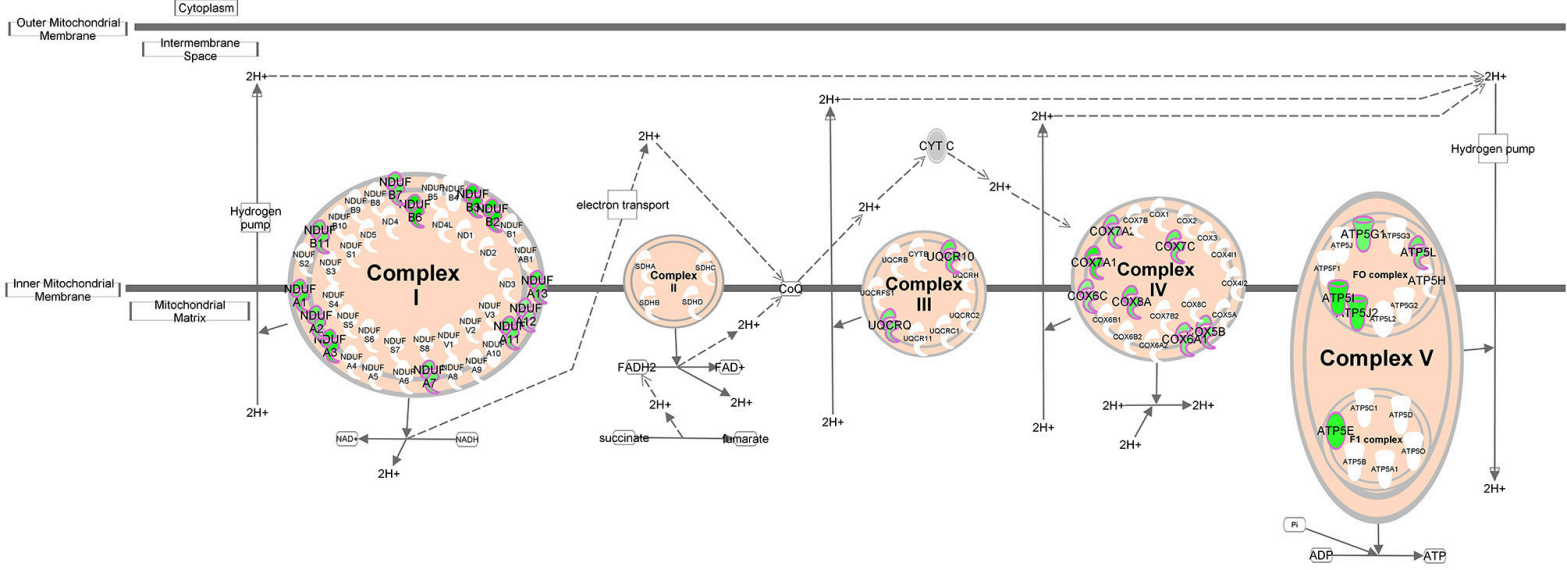
Oxidative phosphorylation pathway. Schematic representation of the oxidative phosphorylation pathway (adapted from Ingenuity). Molecules outlined in purple are differentially expressed within our dataset. Genes in green have decreased expression in *T. gondii* infected cells 6 h post-infection.

As genes work in concert to carry out systemic functions, we used network analysis in IPA to visualize how the differentially expressed genes at 6 h post-infection interacted with each other. Mapping of gene interactions between the 498 genes identified one large network containing 283 genes (57%), with 11 hub genes (i.e., genes which are highly connected) identified (Figure 2); Von Hippel-Lindau Tumor Suppressor (*VHL*), nuclear protein 1, transcriptional regulator (*NUPR1*), hypoxia inducible transcription factor 1 alpha (*HIF-1*α), ISG15 ubiquitin-like modifier (*ISG15*), CD44 molecule (*CD44*), POU class 5 homeobox 1 (*POU5F1*), caspase 8 (*CASP8*), fibronectin 1 (*FN1*), heterogenous nuclear ribonucleoprotein 1 (*HNRNPA1*), MDM2 proto-oncogene (*MDM2*), and ribosomal protein L35 (*RPL35*). Canonical Pathway Analysis was then used to search for classical pathways enriched for genes in the 6 h dataset (Table 1). The top two pathways identified were the inter-related pathways of oxidative phosphorylation (adjusted *p*-value = 6.30 × 10^−19^) (Figure 3) and mitochondrial dysfunction (adjusted *p*-value = 7.94 × 10^−17^). The majority of the differentially expressed genes in these pathways fall within complexes I, III, IV, and V of the OXPHOS pathway and are part of the network in Figure 2.

**Table 1:** List of pathways identified by IPA Canonical Pathway Analysis.

**Pathway**	**BH-adjusted *p*-value**	**Ratio**	**No. of genes in network**	**Genes**
Oxidative phosphorylation	6.30 × 10^−19^	27/109	23	↓*ATP5G1*, ↓***NDUFA7***, ↓***COX6A1***, ↓***COX6C***, ↓***COX5B***, ↓*COX8A*, ↓***ATP5L***, ↓***NDUFA1***, ↓***NDUFB3***, ↓***ATP5E***, ↓***NDUFA2***, ↓***ATP5J2***, ↓***NDUFB6***, ↓***ATP5I***, ↓***COX17***, ↓***COX7C***, ↓*COX7A1*, ↓***NDUFA13***, ↓***NDUFB11***, ↓***NDUFA11***, ↓***UQCR10***, ↓***NDUFB7***, ↓***COX7A2***, ↓***NDUFA12***, ↓***NDUFA3***, ↓***UQCRQ***, ↓*NDUFB2*
Mitochondrial dysfunction	7.94 × 10^−17^	30/171	26	↓***HSD17B10***, ↓***FURIN***, ↓*ATP5G1*, ↓***COX6A1***, ↓***NDUFA7***, ↓***COX6C***, ↓***COX5B***, ↓*COX8A*, ↓***ATP5L***, ↓***NDUFA1***, ↓***ATP5E***, ↓***NDUFA2***, ↓***NDUFB3***, ↓***ATP5J2***, ↓***NDUFB6***, ↑***CASP8***, ↓***ATP5I***, ↓***COX17***, ↓***COX7C***, ↓*COX7A1*, ↓***NDUFA13***, ↓***NDUFB11***, ↓***NDUFA11***, ↓***UQCR10***, ↓***NDUFB7***, ↓***COX7A2***, ↓***NDUFA12***, ↓***NDUFA3***, ↓***UQCRQ***, ↓*NDUFB2*
EIF2 signaling	6.17 × 10^−4^	17/221	17	↓***RPL36A***, ↓***RPS27***, ↓***RPL29***, ↓***RPS21***, ↓***RPS29***, ↓***FAU***, ↓***RPS28***, ↑***RPL27A***, ↓***RPS15***, ↓***RPL35***, ↓***RPS13***, ↓***RPS26***, ↑***HNRNPA1***, ↑***EIF4A1***, ↓***RPS15A***, ↓***RPL36***, ↓***RPL13A***
mTOR signaling	8.1 × 10^−3^	14/199	12	↓*RND2*, ↓***RPS28***, ↓***RPS15***, ↓***RPS26***, ↓***RPS27***, ↓***RPS13***, ↓*PPP2R5B*, ↑***EIF4A1***, ↑***RPS6KA3***, ↓***RPS15A***, ↓***RPS21***, ↑***HIF1A***, ↓***RPS29***, ↓***FAU***
Regulation of eIF4 and p70S6K Signaling	0.036	11/157	10	↓***RPS28***, ↓***RPS15***, ↓***RPS26***, ↓***RPS27***, ↓***RPS13***, ↓*PPP2R5B*, ↑***EIF4A1***, ↓***RPS15A***, ↓***RPS21***, ↓***RPS29***, ↓***FAU***

*Pathways identified at 6 h post-infection by IPA Canonical Pathway Analysis with their respective Benjamini-Hochberg (BH) adjusted p-values, ratios and genes within our dataset which are involved in the listed pathways. Ratios represent number of genes in our dataset out of the number of genes involved in the identified pathway. Up- and downwards facing arrows denote the directionality of the fold change of its adjacent gene. Genes highlighted in bold are part of the network in Figure 2*.

Dysregulation of individual gene expression is caused by upstream regulators such as transcription factors or chemicals exerting their effects upstream of these genes. Evidence for upstream regulator activity on their target genes was investigated (Table 2) to determine if they were related to our identified pathways (Table 1) or network (Figure 2). There were 238 upstream regulators (*p*-value < 0.05) identified for our dataset of which 10 had robust predicted changes in activity with z-score ≤ −2 (= inhibited) or ≥2 (= activated) in *T. gondii* infected cells (Table 2). The most prominent endogenous regulator predicted to be activated is rapamycin-insensitive companion of mammalian target of rapamycin (RICTOR) which targets genes involved in all five pathways identified in Table 1. Other endogenous upstream regulators predicted include two transcriptional regulators; lysine (K)-specific demethylase 5B (KDM5B) (activated) and retinoblastoma 1 (RB1) (inhibited) and endogenous chemical D-glucose (activated) found in mammals (Table 2).

**Table 2:** Upstream regulator activities and their target genes in the dataset at 6 h post-infection.

**Upstream Regulator**	**Molecule Type**	**Predicted activation**	**Activation z-score**	**Overlap *p*-value**	**Target genes in dataset**
RICTOR	Other	Activated	4.83	1.37 × 10^−14^	***ATP5G1, ATP5J2, ATP5L, ATP6V1C1, ATP6V1F, COX17, COX6A1, COX7A1, COX7A2, COX8A**, FAU, *HIF1A, ISG15*, **NDUFA1, NDUFA11, NDUFA2, NDUFA3, NDUFA7, NDUFB2, NDUFB3, NDUFB6, NDUFB7, NDUFC1**, RPL13A, RPS13, RPS15, RPS21, RPS26, RPS29, SHFM1, **UQCR10, UQCRQ***
ST1926	Chemical drug	Activated	3.80	4.78 × 10^−14^	***ATP5I**, C19orf43, CCT6A, **COX5B, COX6C, COX7A2, COX7C**, EIF4A1, KIF3A, NME1, NOP10, RBX1, RPL29, RPL36A, RPS15, RPS26, RPS27, SHFM1, SLIRP, SNRPF, SNRPG, TMEM258, TOMM7, UBL5, UXT*
KDM5A	Transcriptional regulator	Activated	3.61	9.41 × 10^−6^	*ATP6V1F, **COX14, COX17, COX6A1, COX7A2**, FXYD1, MRPL53, NDUFA13, **NDUFA2, NDUFA3**, NME1, TOMM7, **UQCRQ***
RB1	Transcriptional regulator	Inhibited	−3.50	5.70 × 10^−4^	*ATP6V1F, ***CASP8***, CDC25C, COX14, **COX17, COX6A1, COX7A2**, DYNLRB1, *FN1*, FXYD1, KRT10, MRPL53, MT1G**, NDUFA13, NDUFA2, NDUFA3**, NME1, TOMM7, **UQCRQ**, UXT*
CD 437	Chemical drug	Activated	2.689	6.58 × 10^−11^	***ATP5L**, CCT6A, CDC25C, **COX6C, COX7A2, COX7C**, EIF4A1, *HNRNPA1*, KIF3A, NME1, NOP10, RBX1, RPL13A, RPL36A, RPS15, RPS27, SHFM1, SLIRP, SNRPF, SNRPG, TMEM258, TOMM7, UBL5, UXT*
Guanidinopropionic acid	Chemical-endogenous non-mammalian	Inhibited	−2.65	1.19 × 10^−4^	***ATP5G1, NDUFA1, NDUFA11, NDUFA12, NDUFA3, NDUFA7, NDUFB2***
D-glucose	Chemical–endogenous mammalian	Activated	2.61	0.036	*CALD1, CALR, DBI, DYRK1A, FKBP2, *FN1*, HGF, *HIF1A*, IL6ST, LY96, MIF, NAMPT, **NDUFA1, NDUFB3**, NR4A2, PKM, PYCR1, SPHK1, SST*
Bexarotene	Chemical drug	Inhibited	−2.53	0.011	*ADIRF, ANAPC11, CCDC107, **COX6A1, COX8A**, FKBP2, MRPL54, **NDUFB2**, NR4A2, RABAC1*
5-fluorouracil	Chemical drug	Activated	2.11	6.42 × 10^−8^	***ATP5I**, CCT6A, COPS8, **COX6A1, COX6C, COX8A**, EIF4A1, *HNRNPA1, MDM2*, **NDUFA1**, PKM, RPL27A, *RPL35*, RPS13, RPS15A, RPS28, RPS29, SNRPF, SNRPG, TCEB2, UBE2C*
Motexafin gadolinium	Chemical drug	Inhibited	−2.00	1.78 × 10^−5^	*MT1E, MT1G, MT1X, MT2A*

*Upstream regulators predicted to be either activated or inhibited and their target genes within the dataset at 6 h post-infection. Genes highlighted in bold are part of the oxidative phosphorylation or mitochondrial dysfunction pathway. Genes which are underlined are identified hub genes of network (Figure 2)*.

### Mitochondrial morphology following *T. gondii* infection

**Figure 4:**
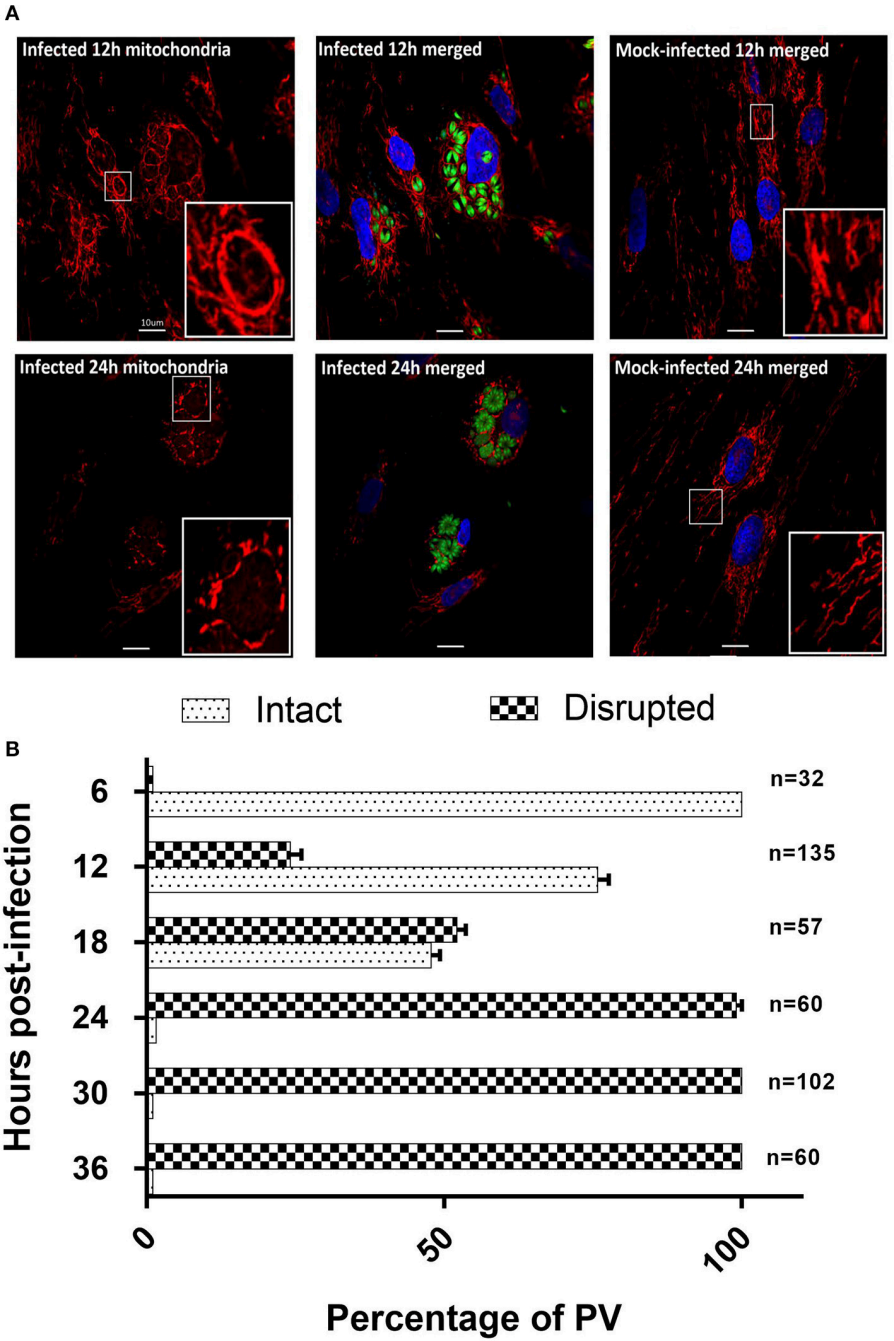
*T. gondii* infected HFF cells reveals changes in morphology at 12 and 24 h post-infection. **(A)** Mitotracker^TM^ Orange (red) and DAPI (blue) to stain the mitochondria and nucleus respectively (See Supplementary Figure 1 for all time points). Results for the 12 h (12 h) and 24 h (24 h) time points are shown here. At 12 h host mitochondria in *T. gondii* infected cells are re-organized and appear as a clear ring around the PVM. At 24 h post-infection host mitochondria around the PVM appear fragmented. Mitotracker^TM^ Orange was also observed staining the mitochondrion of *T. gondii*. Mitochondria in mock-infected cells are structurally different from *T. gondii* infected HFFs at the same time points. Bars represent 10 μM; insert boxes are enlarged mitochondria. **(B)** Percentage ± SEM of the three biological replicate experiments of intact or disrupted mitochondria surrounding the parasitophorous vacuoles from 6 h post-infection to 36 h post-infection. The number of parasitophorous vacuoles quantified per time-point are indicated on the right side of the bar graphs.

Recruitment of host mitochondria to *T. gondii*'s parasitophorous vacuole (PV) has been well-documented (Sinai et al., 1997; Magno et al., 2005; Pernas et al., 2014), occurring as quickly as 10 min post-infection (Sinai and Joiner, 2001). To obtain more detailed information on changes in mitochondrial morphology over time, we used MitoTracker™ Orange together with GFP-expressing *T. gondii* to monitor host mitochondria morphology over 36 h post-infection. When stained with MitoTracker™ Orange, the mitochondria in mock-infected cells presented as long, filamentous organelles concentrating in perinuclear regions and extending out toward the cell membrane (Figure 4). Their morphology did not change over the 36 h. In contrast, *T. gondii* infected cells show strong staining of mitochondria recruited to the PVM of intracellular parasites at 6 h post-infection with associated changes in morphology including apparent enlargement and changes in shape.

The non-PVM-associated mitochondria in infected cells are morphologically similar to the mitochondria in mock-infected cells. At 12 and 18 h post-infection (Figure 4), there is continuous association of mitochondria around the enlarged PVM in which the parasites have multiplied. At the resolution of confocal microscopy it was not possible to determine whether the increase in intensity of mitochondrial staining associated with the PVM was due to increase in size of individual mitochondria or to fusion with additional mitochondria being recruited to the PVM. At these time points, we continued to observe filamentous non-PVM-associated mitochondria in these infected cells. At 24–36 h post-infection the continuous network of mitochondria previously observed surrounding the PVM now presented as fragmented mitochondria, and non-PVM-associated filamentous mitochondria were no longer observed (Figure 4A). We are unable to tell if the fragmented host mitochondria are a result of normal mitochondrial fission in response to the cellular environment or are being selectively degraded as part of mitophagy. To determine the extent of mitochondrial fragmentation, we classified the mitochondria around the PV as either intact or disrupted from three biological replicate infection experiments (Figure 4B). The percentage of intact mitochondria was 100% at 6 h post-infection, 75.8 ± 3.2% at 12 h post-infection, 47.8 ± 2.5% at 18 h post-infection, 2.9 ± 1.7% at 24 h post-infection, and there were no intact mitochondria present at 30 and 36 h post-infection (Figure 4B). Overall results of this analysis confirm previous studies (de Melo et al., 1992; Lindsay et al., 1993; Sinai et al., 1997; Pernas et al., 2014) showing that the parasite has an impact on mitochondrial morphology during infection, and furthermore that this is dynamic over time post-infection including eventual fragmentation of mitochondria which has not previously been reported (See Supplementary Figure 1).

**Figure 5:**
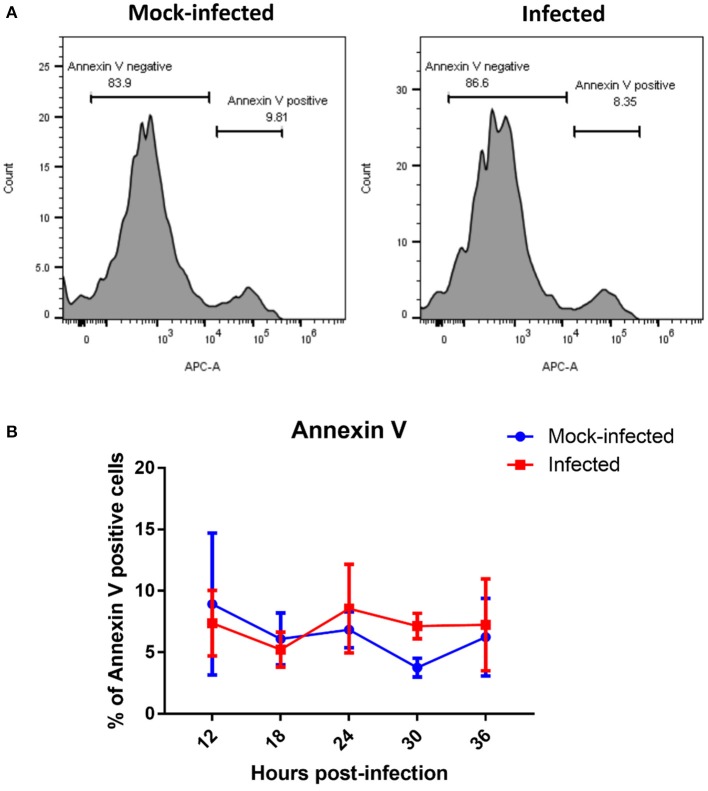
Measurement of apoptotic cells by flow cytometry analysis of Annexin V. **(A)** Representative histograms of mock-infected and infected cells stained with Annexin V at 36 h post-infection showing bimodal distribution of Annexin V negative non-apoptosing cells and positive apoptosing cells. **(B)** Analysis of Annexin-V by flow cytometry revealed no significant difference in percentage of Annexin V positive cells between *T. gondii* mock-infected and infected cells at from 12 to 36 h post-infection. Values represent the mean ± *SD* of one representative experiment with three replicates. Similar results were obtained in two further experiments (data not shown).

The presence of fragmented mitochondria has previously been observed prior to apoptotic cell death (Lee et al., 2004). We therefore determined whether this change in mitochondrial morphology was associated with an increase in apoptosis. However, we did not observe any differences in the rate of apoptosis between infected and mock-infected cells from 12 to 36 h post-infection (Figure 5), suggesting that apoptosis is not responsible for the fragmented mitochondrial morphology observed at later time points in *T. gondii* infected cells.

### Mitochondrial superoxide levels

**Figure 6:**
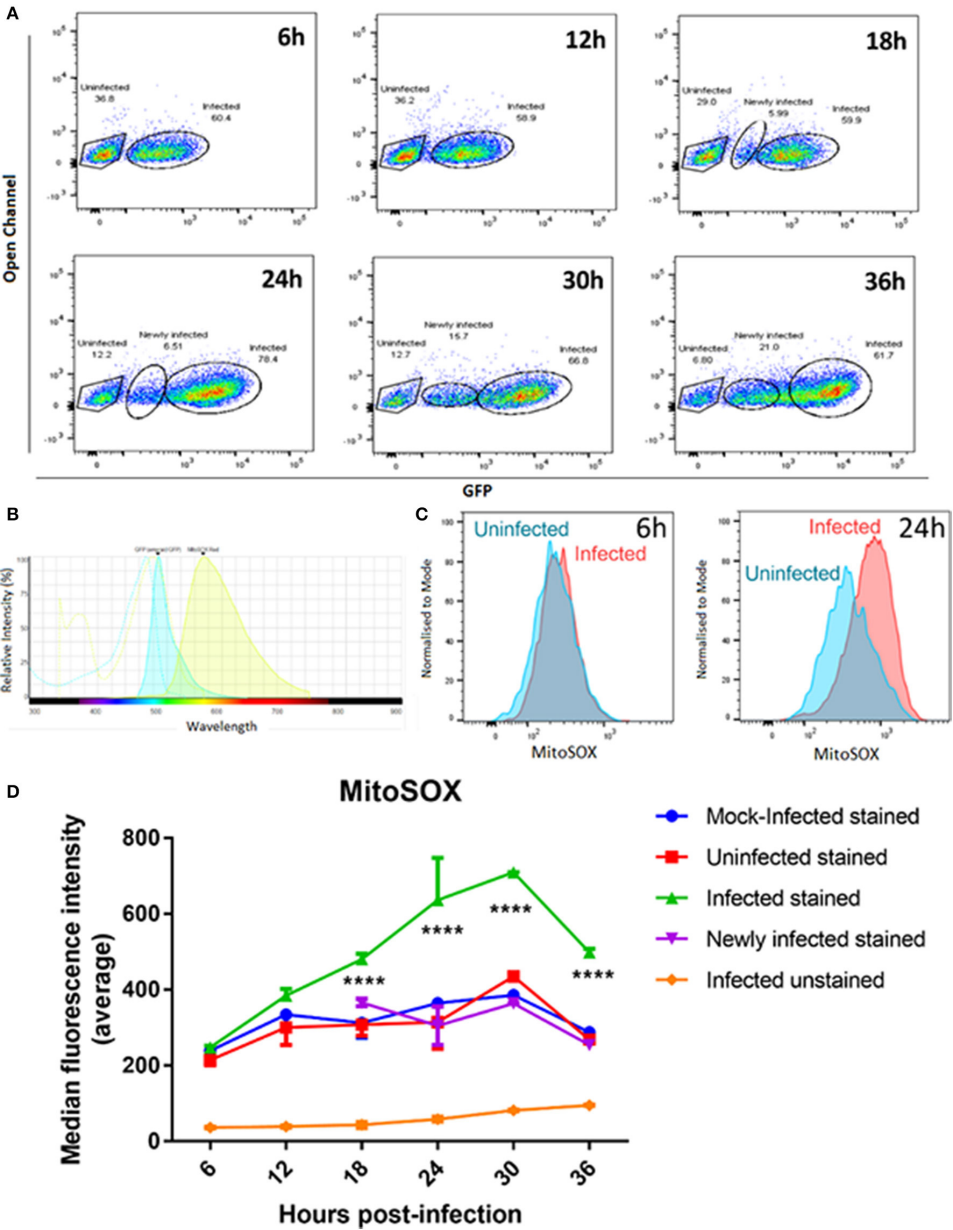
MitoSOX^TM^ staining of mitochondrial superoxide in mock-infected, uninfected, and *T. gondii* infected cells. **(A)** Our gating strategy showing the segregation of uninfected, newly infected and infected cells with replicating parasites within the same population. Cells showing lower GFP fluorescence intensities at later time points were treated as a separate group from the infected cell population as they likely represent cells newly infected with GFP-expressing *T. gondii*. **(B)** Histogram shows the spectra overlap between GFP and MitoSOX^TM^ fluorophores. **(C)** Representative histograms showing MitoSOX^TM^ fluorescence intensities of uninfected and *T. gondii* infected cells. There is a shift of MitoSOX^TM^ fluorescence to the right in infected cells at 24 h post-infection indicating higher levels of mitochondrial superoxide present. **(D)** Significant increases in superoxide levels in infected cells with replicating parasites (^****^adjusted *p*-value < 0.001) compared to either mock-infected, newly-infected or uninfected cells are observed from 18 h post-infection. Values represent the mean values of median fluorescence intensity ± SEM of two experiments with three replicates each.

Mitochondrial oxidative stress can also cause mitochondrial fragmentation (Wu et al., 2011). We therefore measured levels of mitochondrial superoxide as an indicator of oxidative stress over time in *T. gondii* infected and mock-infected cell populations using the mitochondrial-targeted probe MitoSOX^TM^ Red. This assay fluoresces when oxidized by superoxide, and is mitochondrial specific as the fluorophore is targeted to the mitochondria. We measured mitochondrial superoxide over 36 h post infection, sampling every 6 h, and using flow cytometry to compare median fluorescent intensities between three groups of cells: (i) mock-infected cells; (ii) infected cells (cells containing GFP-expressing parasites); and (iii) uninfected cells (cells from infection culture not containing a GFP-expressing parasite). As GFP-expressing parasites replicate within the cells, the fluorescence intensity of GFP increases over time (Figure 6A) making it possible to distinguish on the scatter plots those cells with multiple parasites, representing replicating infection, from newly infected cells (Figure 6A). We therefore gated on these two different infected cell populations to measure mitochondrial superoxide production over the course of infection (Figure 6A).

As there is a small overlap in fluorescence spectrum between GFP and MitoSOX^TM^ fluorophores (Figure 6B) we compared the MitoSOX^TM^ fluorescence in both infected and mock/uninfected cells not stained with MitoSOX^TM^ to determine if the increasing number of GFP-expressing parasites in the cells affected MitoSOX^TM^ fluorescence. There was no significant difference in MitoSOX^TM^ fluorescence intensities between unstained cells infected with GFP-expressing parasites (Figure 6D) and unstained mock- or unstained uninfected cells (data not shown). Thus, bleed-through of GFP fluorescence does not contribute to an increase in MitoSOX^TM^ fluorescence. Uninfected cells behaved in a similar manner to mock-infected cells, i.e., levels of superoxide did not differ significantly between them throughout the 36 h time course (Figure 6D). The newly infected cell population, measured at 18, 24, 30, and 36 h post-infection, also showed no significant difference in superoxide production compared to uninfected cells. This indicates that exclusion of newly infected cells from the infected cell population with replicating parasites observed at the later time points is necessary for accurate measurements of mitochondrial superoxide over the course of infection. In contrast, infected cells with replicating parasites showed a steady increase in superoxide levels post-infection, rising to a 2.5-fold increase at 30 h post-infection before declining at 36 h. Figure 6C shows there is a larger difference in MitoSOX^TM^ staining in *T. gondii* infected cells at 24 h post-infection compared to uninfected cells than at 6 h post-infection.The levels of superoxide in infected cells with replicating parasites are significantly higher than mock-infected and uninfected cells at 18, 24, 30, and 36 h (Figure 6D). Since we are measuring uninfected cells, newly infected, and cells with established replicating parasites in the same population, these results indicate that stimulation of superoxide is dependent on active parasite invasion and replication of parasites, i.e., that superoxide generation requires intracellular establishment of *T. gondii* and is not stimulated by factors secreted by the parasite or neighboring infected cells. It is also possible that MitoSOX^TM^ can stain *T. gondii-*derived superoxide in addition to human mitochondrial superoxide. However, the fact that the highest parasite load occurs concurrent with the maximum decrease in levels of MitoSOX^TM^ at 36 h post-infection (Figure 6D) suggests that parasite-derived superoxide is not contributing significantly to the result.

### OXPHOS complex protein levels in infected cells

OXPHOS is a process that generates high amounts of energy in the form of adenosine triphosphate (ATP) and is the major contributor of mitochondrial superoxide production. It occurs on the inner membrane on the mitochondria through the four complexes of the electron transport chain (ETC) creating a proton gradient that powers ATP synthase to generate ATP from adenosine diphosphate (ADP) and phosphate. Our gene expression microarray revealed an enrichment of genes with decreased expression in OXPHOS complexes I, III, IV, and V (Figure 3) following *T. gondii* infection. To determine whether this translated to an effect at the protein level which would subsequently play a role in increased superoxide production observed in *T. gondiii* infected cells, we compared the protein levels of all five complexes between infected and mock-infected cells at the same infection time points as the MitoSOX experiments.

**Figure 7:**
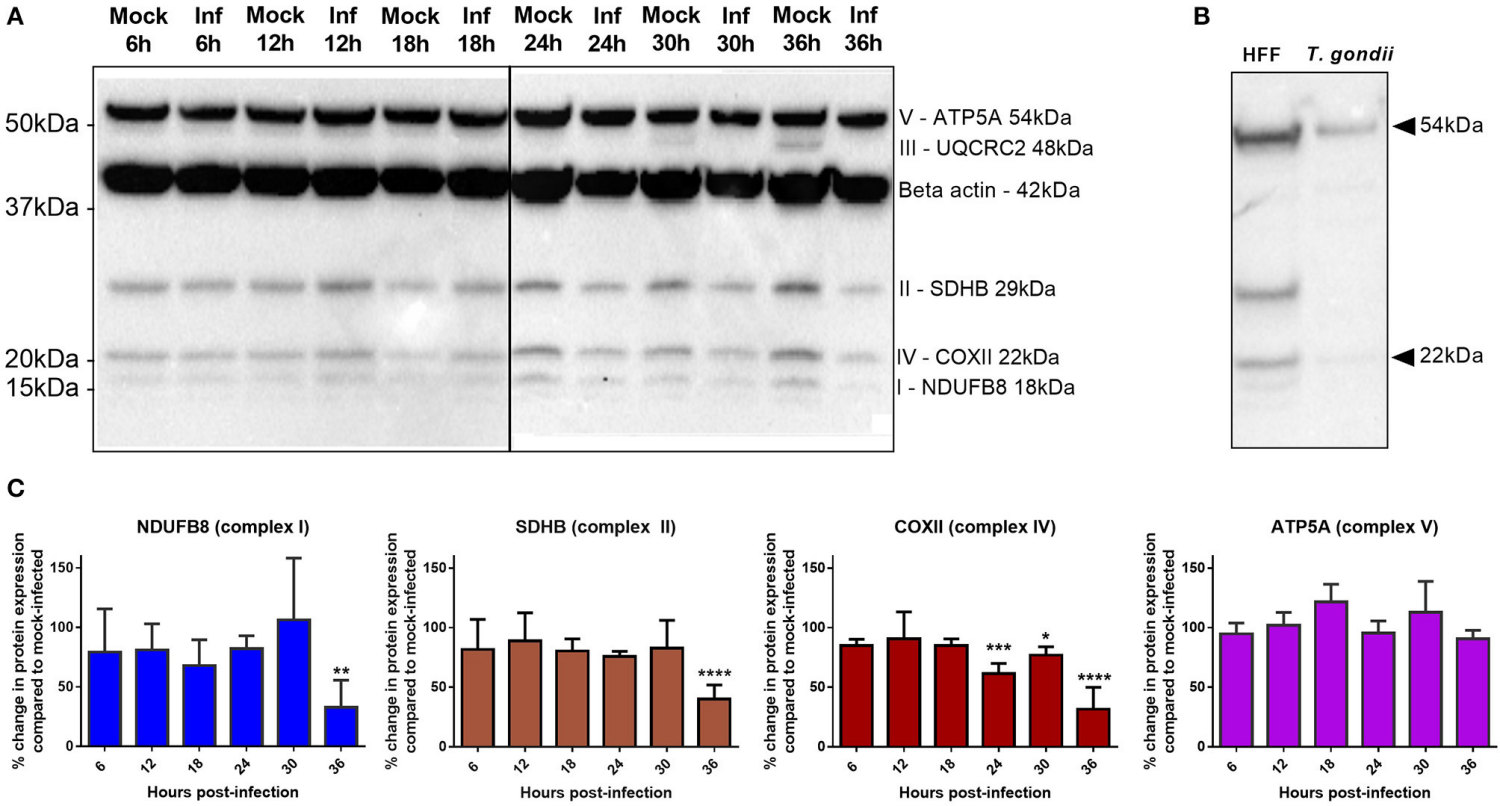
Comparison of OXPHOS protein expression between *T. gondii* infected cells and mock-infected cells. **(A)** Western blots of infected and mock-infected cells stained with OXPHOS antibody cocktail and β-actin antibody. Protein bands from complexes I, II, IV, and V and β-actin are consistently detected in our sample while complex III is only detected in mock-infected cells at 30 and 36 h time points in this experiment, and not detected in two repeat experiments (not shown). **(B)** Western blot of uninfected HFF cells and purified *T. gondii* parasites stained with OXPHOS antibody cocktail only (no β-actin antibody staining). Presence of faint bands detected at the expected sizes of human Complex IV and V, suggesting host cell contamination. No other bands were present to represent parasite cross-reactivity. **(C)** Quantitative analysis of the western blots using densitometry (normalized to β-actin) show a significant decrease in complex I and II at 36 h post-infection and a decrease in complex IV from 24 h post-infection. No significant changes in complex V proteins were observed over the time course of these experiments. Values represent mean ± *SD* of three experiments. (^*^adjusted *p*-value = 0.0352 ^**^adjusted *p*-value = 0.0052, ^***^adjusted *p*-value = 0.0002, ^****^adjusted *p*-value = <0.0001).

Western blot analysis was performed using an anti-OXPHOS antibody cocktail targeted to proteins in the different complexes. We were unable to consistently detect the protein band for complex III in our samples (Figure 7A). To investigate whether the anti-OXPHOS antibody cocktail had cross-reacted with parasite mitochondrion, we performed a western blot analysis of preparations of purified proteins from isolated *T. gondii* parasites. Faint bands were present at the expected sizes for human Complex IV and V which we interpreted as host cell contamination (Figure 7B). As *T. gondii* are obligate intracellular parasites, protein extraction of purified parasites depended on lysis of infected host cells. Though we used a 5 μm filter to remove host cell debris, host cell fragments may still pass through. No other bands were present that might represent parasite-derived cross-contamination (Figure 7B). As we were unable to definitively rule out cross-species contamination, we checked the protein sequences of the five OXPHOS complex against the *Toxoplasma* genome using BLAST and there were no homologous sequences within the whole Toxoplasma genome which suggests that these mitochondrial probes have no cross-reactivity.

Results show that the protein levels of complexes I, II, IV, and V remain stable up to 18 h post-infection with *T. gondii*; no significant difference in protein levels is detected up to this time point between infected and mock-infected cells (Figure 7C) or between infected and flow sorted uninfected cells (data not shown). However, from 24 h post-infection complex IV shows a decrease in protein level in infected relative to mock-infected cells, with complexes I and II also showing a significant decrease in protein level at 36 h post-infection. Complex V does not significantly differ in protein level between mock-infected and infected cells at any time point post-infection (Figure 7C).

### Mitochondrial membrane potential

**Figure 8:**
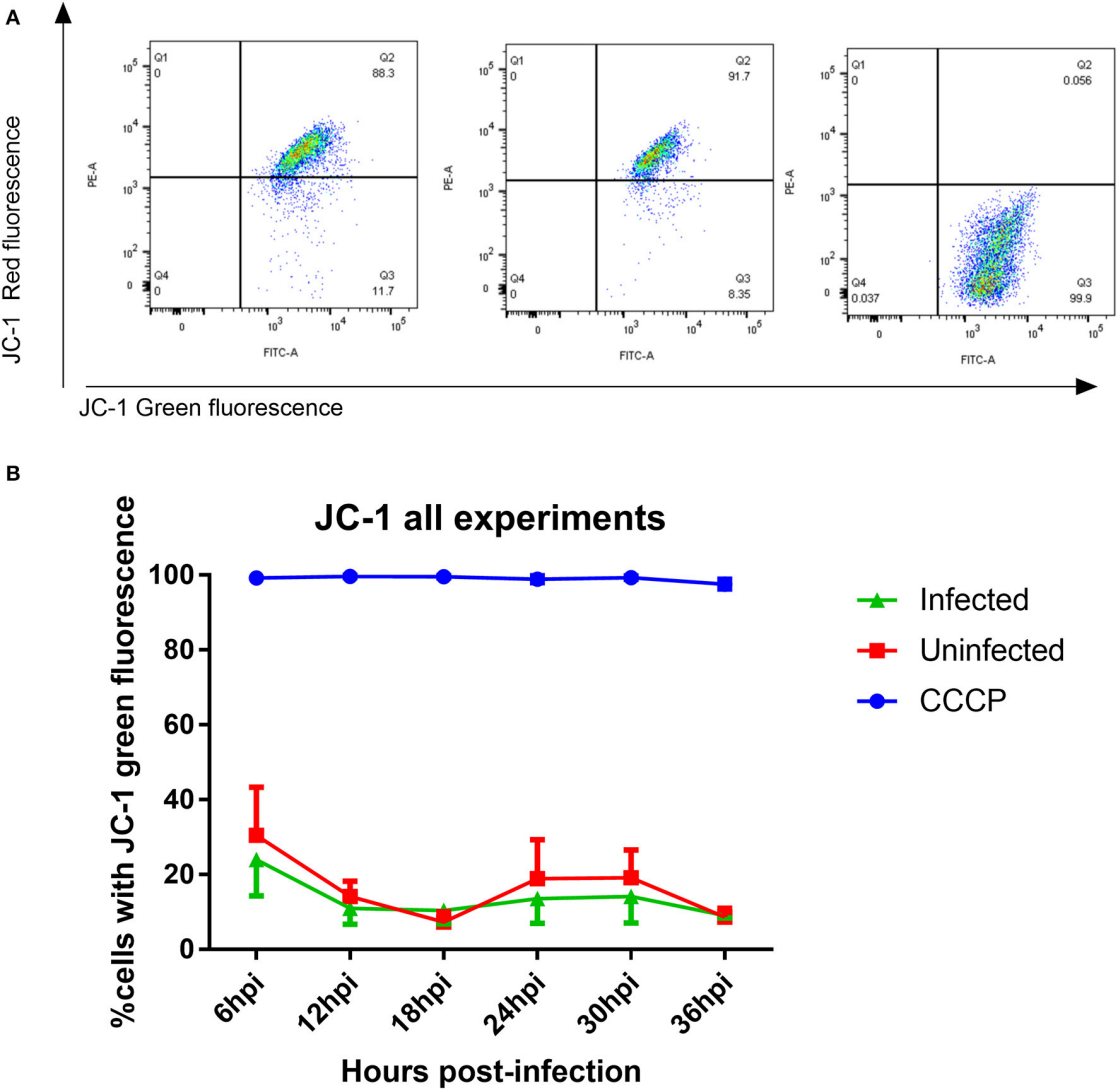
Measurement of mitochondrial membrane potential using JC-1 dye **(A)** JC-1 analysis of mock-infected and *T. gondii* infected cells. Changes in red vs. green fluorescence were used to measure mitochondrial membrane potential following infection. CCCP-treated cells were used as positive controls to establish gating strategies. Cells which appear in quadrant 3 represent cells with depolarized mitochondrial membranes. **(B)** Representative figure showing the mitochondrial membrane potential was stable over time in both infected (green) and mock-infected cells (red). There was no significant difference in mitochondrial membrane potential between infected and uninfected cells. One hundred percent of cells treated with CCCP (blue) had depolarized mitochondrial membranes. Values represent mean percentage of cells with depolarized mitochondrial membrane ± SEM of three experiments with three replicates each.

OXPHOS through the ETC and ATP synthase is reliant on a hyperpolarized mitochondrial membrane potential (MMP) so measurement of MMP allows us to determine if a change in MMP is the underlying cause for decreased OXPHOS protein expression. Using the dual fluorescent JC-1 dye, we could determine whether infection leads to a depolarization of MMP (a shift from red to green fluorescence) using flow cytometry. The drug CCCP, which permeabilizes the inner mitochondrial membrane (Minamikawa et al., 1999) leading to MMP depolarization, was used as a positive control to set the gates for depolarized MMP (Figure 8A). No significant changes were observed in the number of cells with depolarized MMP between infected and mock-infected cells at any time point between 6 and 36 h post-infection (Figure 8B). The MMP of both experimental groups remained steady over time.

## Discussion

Although host mitochondrial association to *T. gondii*'s PVM has been described for several decades, studies on how *T. gondii* interacts with the host mitochondria and the role it plays during infection are only recently emerging. The impact of this rapid, strain-specific association is revealed by proteomic (Nelson et al., 2008) and transcriptomic studies (Blader et al., 2001; He et al., 2016), including the present study, showing perturbations of mitochondrial metabolic pathways such as glycolysis (Blader et al., 2001) and OXPHOS [(He et al., 2016) and our study] in *T. gondii* infected cells. Host mitochondrial association has also been associated with transcriptional changes of host innate immunity (Pernas et al., 2014). Since *T. gondii* closely interacts with the host mitochondria during infection, we wanted to study in more detail the functional changes of host mitochondria in the acute stages of infection.

Mitochondria undergo continual fission and fusion to maintain their morphology and function in response to stimuli (Chauhan et al., 2014). We therefore examined in detail the changes in host mitochondrial morphology that occurred over the course of a 36 h infection *in-vitro*. Consistent with previous studies (Sinai et al., 1997; Sinai and Joiner, 2001; Pernas et al., 2014), we observed recruitment and apparent enlargement of mitochondria to the PVM early in the infection. However, as infection progressed beyond 24 h, we observed the presence of fragmented host mitochondria surrounding the PVM that has not previously been reported. This likely reflects the fact that no previous studies have followed mitochondrial changes for more than 24 h post-infection. Mitochondrial fragmentation has been linked to cellular apoptosis (Lee et al., 2004) but our Annexin V assay showed no increase in cells undergoing apoptotic death following *T. gondii* infection. Since mitochondrial morphology is clearly impacted by *T. gondii* infection, we undertook further studies to determine whether there were effects on mitochondrial function.

Mitochondrial superoxide production, OXPHOS, and mitochondrial membrane potential are intrinsically linked. Electrons transferred through the ETC comprising complexes I to IV generate the mitochondrial proton motive force across the inner mitochondrial membrane used to power ATP synthase (also known as Complex V) to generate ATP from ADP and phosphate. The proton motive force consists of two components; the mitochondrial membrane potential, which represents the major portion of the proton motive force, and the pH difference across the inner mitochondrial membrane. The proton motive force regulates the activity of the ETC complexes: high membrane potential inhibits further proton pumping, whereas a decrease of proton motive force through proton utilization e.g., by ATP synthase, would allow the ETC to re-establish the proton motive force (Hüttemann et al., 2008). During the process of OXPHOS, electrons may leak from the ETC Complexes I and III and react with oxygen to form superoxide. In our study, we observed an increase in mitochondrial superoxide production without the concomitant increase in OXPHOS complexes or changes in mitochondrial membrane potential usually observed in OXPHOS. This suggests that the increase in superoxide production may not be due to an increase in aerobic respiration through OXPHOS. This is supported by our transcriptomic data indicating a down-regulation of OXPHOS following *T. gondii* infection and our western blot analyses showing decreased protein expression of Complex IV at 24–36 h post-infection, and decreases in Complexes I and II at 36 h post-infection.

Conversely, decreased OXPHOS has also been shown to increase superoxide levels (Dawson et al., 1993; Batandier et al., 2006; Murphy, 2009). A reduced ETC may stimulate superoxide formation by Complex III by favoring reverse electron transfer from Complex II to Complex I (Batandier et al., 2006; Murphy, 2009) and inhibition of Complex IV enhances superoxide generation by increased reduction of redox centers in complex I or complex III promoting electron leak and ROS generation from these complexes (Dawson et al., 1993). A down-regulation of OXPHOS is usually accompanied by increased cellular glycolysis, the process of breaking down glucose into pyruvate, which feeds into the citric acid cycle producing substrates such as NADH and FADH for OXPHOS complexes to generate ATP. A high glycolytic flux, which is the rate at which molecules pass through glycolysis, has been associated with enhanced superoxide production (Zhou et al., 2005). Several studies have shown increased host glycolysis following *T. gondii* infection (Blader et al., 2001; Nelson et al., 2008; Menendez et al., 2015) which is regulated by host HIF-1α (a hub gene in our network) and hexokinase 2 (HK2) (Menendez et al., 2015). Activation of HIF-1α and HK2 is dependent on mTOR complex 2 (mTORC2) (Toschi et al., 2008; Betz et al., 2013), previously shown to play a central role in mitochondrial distribution following *T. gondii* infection (Wang et al., 2010). Consistent with this, we observed enrichment of differentially expressed genes in the mTOR pathway, while RICTOR, a component of mTORC2, was the top predicted endogenous upstream regulator with increased activity. RICTOR knockdown leads to an increase in mitochondrial respiration (Schieke et al., 2006) and dysregulation of mTORC2 is associated with mitochondrial defects (Betz et al., 2013). Through Akt signaling, mTORC2 promotes HK2 phosphorylation of glucose stimulating glycolysis (Hagiwara et al., 2012). HK2 is also associated with switching cellular metabolism from OXPHOS to glycolysis (Wolf et al., 2011).

Given the down-regulation of OXPHOS complexes in *T. gondii* infected cells, it is of interest to consider how the mitochondrion is maintaining its ETC-reliant membrane potential. The glycolysis process itself generates ATP but with much less efficiency than OXPHOS. In cells that rely heavily on aerobic glycolysis instead of OXPHOS for energy, glycolytic ATP is essential for maintaining the mitochondrial membrane potential and preventing apoptotic death (Chevrollier et al., 2011). Adenine nucleotide translocator 2 (ANT2) imports glycolytic ATP into the mitochondria which the reversible proton pump ATP synthase (OXPHOS Complex V) then hydrolyses to pump protons back into the inner mitochondrial membrane to sustain the mitochondrial membrane potential (Chevrollier et al., 2011). This could explain the lack of change in mitochondrial membrane potential and ATP synthase protein expression in *T. gondii* infected cells we observed despite increased levels of superoxide production and a down-regulation of ETC protein expression.

As glycolytic flux is regulated by glucose uptake, we hypothesize that there is increased glucose uptake in a *T. gondii* infected cell. This would be consistent with our observation that D-glucose was a strongly predicted endogenous upstream regulator, influencing expression of multiple genes including the hub genes *FN1* and *HIF-1*α. Exposing cells to high glucose *in-vitro* has been shown to induce mitochondrial fragmentation by fission through glucose-dependent ROS overproduction (Yu et al., 2006). This, in turn, may account for the fragmented mitochondria seen occurring from 24 h post-infection. It was previously proposed that a change in mitochondrial morphology is required to produce more ROS, possibly due to a bigger relative membrane surface (Yu et al., 2006). In recent years, ROS production has been shown to play a role in signaling between mitochondria and other cellular processes in response to stress (Sena and Chandel, 2012). However, when production of ROS is left unchecked, this can lead to oxidative stress and, in turn, mitochondrial dysfunction.

Recently, maternal infection with *T. gondii* Wh3 isolate of genotype Chinese 1 strain was observed to induce mitochondrial dysfunction associated trophoblast apoptosis in pregnant mice (Xu et al., 2015). Of interest, transcriptomic analysis of mitochondria-DNA depleted breast epithelial cells and normal cells undertaken to understand pathogenesis of breast tumorigenesis (Kulawiec et al., 2008) revealed increased expression of *FN1*, which was also the hub gene of one of the major gene networks identified in their study. The observation of *FN1* as a focal gene in seemingly unrelated diseases suggests that *FN1* may have a role in the pathogenesis of diseases stemming from dysfunctional mitochondria. On a systemic level, mitochondrial dysfunction can affect multiple organs in the body but the organs most affected are those which require the highest mitochondrial activity such as the eye, brain, and muscle. These organs have large numbers of mitochondria to cope with their increased cellular metabolic needs. Mitochondrial dysfunction has been linked to ophthalmic, neurological, and muscular diseases such as Leber's hereditary optic neuropathy (Brown, 1999) and Leigh's syndrome (Thorburn et al., 1993–2017). This is of interest in relation to our study as *T. gondii* preferentially infects the eye, brain and muscles. The eye and brain are also sites of clinical manifestations following *T. gondii* infection; for example, the classic triad of retinochorioditis, hydrocephalus, and intracranial calcifications in congenital toxoplasmosis, and toxoplasmic encephalitis in immunosuppressed patients with acquired toxoplasmosis. Mitochondrial dysfunction is also associated with neurodegenerative diseases such as Parkinson's disease (Miman et al., 2010), Alzheimer's disease (Kusbeci et al., 2011), and schizophrenia (Prabakaran et al., 2004). Again, this is of interest as associations between *T. gondii* infection and Parkinson's, Alzheimer's disease (Kusbeci et al., 2011; Jung et al., 2012) and schizophrenia (Wang et al., 2013) have been proposed. These findings suggest that the disruption of host mitochondria may play a role in the pathogenesis of toxoplasmosis and clinical signs observed in congenital and acquired infections.

In summary, our transcriptomic data, supported by the characterization of host mitochondrial parameters such as morphology, superoxide production, OXPHOS protein levels, and membrane potential, suggest a perturbation of the host mitochondrial processes following *T. gondii* infection. It is unclear whether these modified mitochondrial functions occur as a consequence of the host response to *T. gondii* or direct manipulation by the parasite. Previous transcriptional (Ngô et al., 2017) and functional studies (Pernas et al., 2014) have indicated that the effects on host mitochondria following infection are strain-specific. Current literature (Xu et al., 2015) and our study have reported perturbations in host mitochondrial functions of experimental models infected only with the Type I mouse-virulent strains. Thus, further studies should be carried out to characterize the host mitochondrial functions of cells infected with other *T. gondii* strains. Mitochondria with decreased functions have been implicated in ophthalmological and neural disease, including neurodegenerative and psychiatric disorders, thus providing new avenues for research into the clinical signs of both congenital and acquired toxoplasmosis.

## Author contributions

GS, DA, JB, and SJ: Conceived and designed the experiments; GS: Performed the experiments; GS, DA: Analyzed the data; GS, DA, JB, and SJ: Wrote the paper.

### Conflict of interest statement

The authors declare that the research was conducted in the absence of any commercial or financial relationships that could be construed as a potential conflict of interest.

## Supplementary material

Supplementary Figure 1 *Toxoplasma gondii* infected cells show distinct morphology changes when compared to mock-infected cells at their respective time-points. HFF cells were infected with GFP-expressing *T. gondii* (green) for 36 h and labeled with Mitotracker TM Orange (red) and DAPI (Blue) to stain the mitochondria and nucleus, respectively.

Supplementary Table 1 List of genes that are differentially expressed at each time-point. Genes that have an adjusted *p*-value of <0.05 and a fold change >1.5 are deemed to be differentially expressed. Genes highlighted in yellow play a role in inter-related pathways oxidative phosphorylation and mitochondrial dysfunction.
