# Supplementary figures and images for: Toxoplasma gondii Infection Is Associated with Mitochondrial Dysfunction in-Vitro
Source: Front Cell Infect Microbiol. 2017 Dec 12;7:512. doi: 10.3389/fcimb.2017.00512 (PMC5733060; doi:10.3389/fcimb.2017.00512)

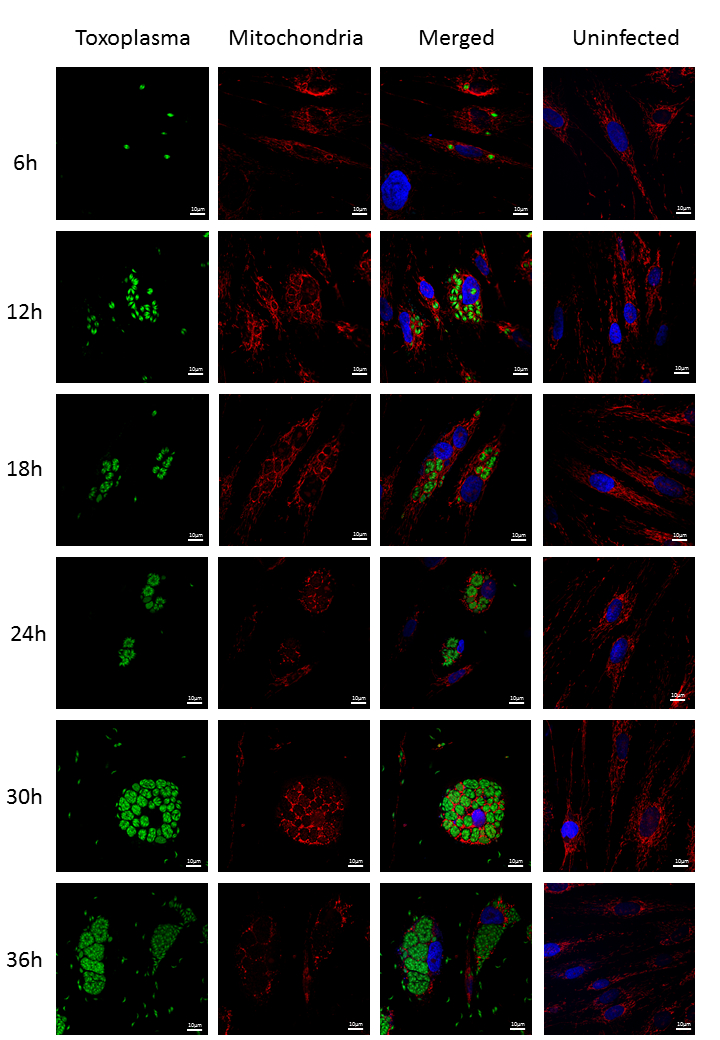

Supplement: Supplementary Figure 1 — Toxoplasma gondii infected cells show distinct morphology changes when compared to mock-infected cells at their respective time-points. HFF cells were infected with GFP-expressing T. gondii (green) for 36 h and labeled with Mitotracker TM Orange (red) and DAPI (Blue) to stain the mitochondria and nucleus, respectively. [file Image1.JPEG]
